# Combined physical training protects the left ventricle from structural and functional damages in experimental pulmonary arterial hypertension

**DOI:** 10.1186/s40885-024-00270-z

**Published:** 2024-05-01

**Authors:** Luciano Bernardes Leite, Leôncio Lopes Soares, Alexandre Martins Oliveira Portes, Thayana Inácia Soares, Bruna Aparecida Fonseca da Silva, Taís Rodrigues Dias, Sebastião Felipe Ferreira Costa, Luiz Otávio Guimarães-Ervilha, Mirian Quintão Assis, Victor Neiva Lavorato, Albená Nunes da Silva, Mariana Machado-Neves, Emily Correna Carlo Reis, Antônio José Natali

**Affiliations:** 1 Department of Physical Education, Laboratory of Exercise Biology, Federal University of Viçosa, Viçosa, Brazil; 2 Department of Biological Sciences, Federal University of Ouro Preto, Ouro Preto, Brazil; 3 Department of General Biology, Laboratory of Structural Biology, Federal University of Viçosa, Viçosa, Minas Gerais Brazil; 4 Department of Physical Education, Governador Ozanam Coelho University Center, Ubá, Minas Gerais Brazil; 5 Physical Education School, Federal University of Ouro Preto, Ouro Preto, Brazil; 6 Veterinary Department, Federal University of Viçosa, Viçosa, Minas Gerais Brazil

**Keywords:** Pulmonary arterial hypertension, Physical training, LV myocyte contractility, Adverse remodeling, Oxidative stress

## Abstract

**Background:**

Under the adverse remodeling of the right ventricle and interventricular septum in pulmonary arterial hypertension (PAH) the left ventricle (LV) dynamics is impaired. Despite the benefits of combined aerobic and resistance physical trainings to individuals with PAH, its impact on the LV is not fully understood.

**Objective:**

To test whether moderate-intensity combined physical training performed during the development of PAH induced by MCT in rats is beneficial to the LV’s structure and function.

**Methods:**

Male Wistar rats were divided into two groups: Sedentary Hypertensive Survival (SHS, *n* = 7); and Exercise Hypertensive Survival (EHS, *n* = 7) to test survival. To investigate the effects of combined physical training, another group of rats were divided into three groups: Sedentary Control (SC, *n* = 7); Sedentary Hypertensive (SH, *n* = 7); and Exercise Hypertensive (EH, *n* = 7). PAH was induced through an intraperitoneal injection of MCT (60 mg/kg). Echocardiographic evaluations were conducted on the 22nd day after MCT administration. Animals in the EHS and EH groups participated in a combined physical training program, alternating aerobic (treadmill running: 50 min, 60% maximum running speed) and resistance (ladder climbing: 15 climbs with 1 min interval, 60% maximum carrying load) exercises, one session/day, 5 days/week for approximately 4 weeks.

**Results:**

The physical training increased survival and tolerance to aerobic (i.e., maximum running speed) and resistance (i.e., maximum carrying load) exertions and prevented reductions in ejection fraction and fractional shortening. In addition, the physical training mitigated oxidative stress (i.e., CAT, SOD and MDA) and inhibited adverse LV remodeling (i.e., Collagen, extracellular matrix, and cell dimensions). Moreover, the physical training preserved the amplitude and velocity of contraction and hindered the reductions in the amplitude and velocity of the intracellular Ca^2+^ transient in LV single myocytes.

**Conclusion:**

Moderate-intensity combined physical training performed during the development of MCT-induced PAH in rats protects their LV from damages to its structure and function and hence increases their tolerance to physical exertion and prolongs their survival.

**Supplementary Information:**

The online version contains supplementary material available at 10.1186/s40885-024-00270-z.

## Background

Pulmonary arterial hypertension (PAH) is a chronic debilitating illness in which the increase in pulmonary vascular resistance and the remodeling of the pulmonary artery induces pressure-overload in the right ventricle (RV) ending thus in pathological rebuilding and further heart failure [1, 2]. The underlying mechanisms of PAH include important oxidative stress in lung and heart that leads to tissue injury and contributes to the pathophysiology of heart failure in humans [3, 4]. In the animal model of monocrotaline (MCT)-induced PAH, the bioactive metabolite of MCT selectively injures the vascular endothelium of lung vessels, worsens the endothelium-dependent nitric oxide (NO)-related relaxation, and augments the levels of endothelin-1 resulting in progressive vasculitis, vascular resistance, arterial pressure and then RV overload, hypertrophy, dilation, and failure [5–7]. In this model a role of oxidative stress in the pathogenesis of RV dysfunction is suggested as MCT increases the production of reactive oxygen species (ROS) and activate proapoptotic pathways [7–12]. In addition, high levels of ROS may activate matrix remodeling and increase fibrosis [13], and lead to contractile dysfunction by directly affecting calcium cycling regulatory proteins [14, 15].

Because of the ventricular interdependence, the RV hypertrophy and dilation and the interventricular septum flattening occurring in PAH negatively affect the left ventricle (LV) structure and function. Individuals with PAH exhibit altered LV geometry (i.e., D-shaped) [4] and consequent impaired filling and reduced end-diastolic volume [16–18]. These changes weaken their LV function as it influences LV segmental function and torsion and decreases the systolic volume and thus their capacity to tolerate physical effort [3, 4]. In experimental PAH induced by MCT the LV presents reduced mass and cell dimensions [5, 8, 16] along with increased collagen and fibrosis [19–21]. Such structural changes lead to LV contractile dysfunctions characterized by reduced ejection fraction and fractional shortening and impaired LV myocyte Ca^2+^ cycling and contractility (i.e., reduced amplitude of contraction and prolonged contraction and relaxation velocities) [20–23].

Regarding treatments for PAH, the main therapies aim to diminish pulmonary arterial pressure to maintain the cardiac function and thus improve the patients’ prognosis, quality of life and survival [24]. In this sense, notably physical exercise has been proven to preserve cardiac function in patients with PAH [25–28]. Furthermore, combined exercise (i.e., aerobic plus resistance) programs of low- to moderate-intensity have been employed and provided significant enhancements in the muscle power, exercise capacity, and survival of individuals with PAH [27, 29, 30]. In the MCT-induced PAH, aerobic and resistance exercise training of low- to moderate-intensity performed independently enhances tolerance to physical effort and survival, mitigates RV adverse remodeling and impaired myocyte Ca^2+^ cycling and contractile function [12, 31–33]. Considering the LV, few studies have tested the effects of exercise training. To illustrate, Schmidt et al. [21] reported that 4 weeks of moderate-intensity aerobic exercise prevents LV dysfunctions, reductions in the cross-sectional area of myocytes and increases in fibrosis. In addition, we demonstrated recently that moderate-intensity resistance exercise training preserves ejection fraction and fractional shortening, mitigates damages to LV cell contractility, and inhibits fibrosis and type I collagen increases [20]. Despite that, the effects of combined exercise training regimes on the LV structure and function in the model of MCT-induced PAH are not known. Therefore, this study was undertaken to investigate whether combined physical training of moderate intensity performed during the development of PAH induced by MCT in rats is beneficial to the LV’s structure and function. We hypothesized that moderate-intensity combined exercise training performed during the development of experimental PAH protects the LV from the structural and functional damages inherent to the model.

## Methods

### Experimental design

Male Wistar rats (Body weight: ~ 200 g) were housed in transparent polycarbonate cages (i.e., 4 animals per cage). Initially, to evaluate survival the rats were randomly divided into two groups: sedentary hypertensive survival (SHS, *n* = 7) and exercise hypertensive survival (EHS, *n* = 7). Subsequently, to evaluate the effects of combined physical training another set of rats were randomly divided into three experimental groups: sedentary control (SC, *n* = 7), sedentary hypertensive (SH, *n* = 7), and exercise hypertensive (EH, *n* = 7). All animals were maintained in a controlled environment at around 22 °C and relative humidity of ~ 60%. They were housed under a 12/12 h light/dark cycle and had access to water and standard rodent chow. Ethical guidelines for animal experimentation were strictly followed. The study was approved by the Ethics Committee on the Use of Animals of the Federal University of Viçosa (CEUA-UFV) under protocol nº 02/2021.

### PAH induction

Animals in the hypertensive groups were administered a single intraperitoneal injection of 60 mg/kg body weight MCT (Sigma-Aldrich, St. Louis, MO, USA) dissolved in saline (140 mM NaCl, pH 7.4) to induce PAH [24, 34]. The control animals received an equivalent volume of saline (140 mM NaCl, pH 7.4).

### Combined physical training protocol

The combined physical training consisted of moderate-intensity aerobic running on an electric treadmill and resistance climbing on a vertical ladder. Exercise training sessions were performed 5 days/week, one session/day over a 21-day period, being each exercise model performed on alternate days. The treadmill running session was composed of 5 min warming up (speed: 5 m/min), 50 min running (speed: 60% of maximum running speed—MRS), and 5 min cooling down (speed: 5 m/min). The resistance exercise of ladder climbing was composed of 15 climbs interspersed with 1 min interval (Load: 60% maximum carrying load—MCL). To determine the exercise intensities maximal tests were previously carried out (see below).

### Maximum running speed test

The rats underwent a 2-week familiarization period with the electric treadmill (AVS Projetos®, São Paulo, Brazil) prior to MCT administration. Following this adaptation phase, a MRS test was conducted. The MRS was evaluated using a progressive treadmill exercise protocol as previously outlined [35]. Initially, the running speed was set at 5 m/min and increments of 3 m/min were implemented every 3 min, with no inclination until fatigue. A point of fatigue was defined, and the test was concluded when the animals were unable to maintain the pace of the treadmill. The MRS and the total running time of each rat were recorded. This evaluation was carried out on two occasions: first, 2 days after the animals became accustomed to the equipment; and second, on day 19 following MCT administration.

### Maximum carrying load test

The rats were introduced to the exercise protocol over a span of two weeks, which involved climbing a vertical ladder [36] carrying a load proportional to their body weight in an apparatus attached to the tail. This adaptation period preceded MCT administration. After the adaptation phase, a MCL test was conducted. The test involved the rat initially climbing a ladder carrying a load equivalent to 75% of his body weight. After successfully completing the ascend and a subsequent rest interval of 120 s, an additional 30-g weight was added for the subsequent climb. This process was repeated iteratively until the rat was unable to ascend the top of the ladder. The highest weight the rat successfully climbed was designated as the MCL. The MCL test was performed on two occasions: first, 4 days after the animals became accustomed to the equipment; and second, on day 21 following MCT administration.

### Echocardiography

Echocardiographic evaluations were carried out on day 22 after the administration of MCT. The procedure involved anesthetizing the animals to ensure their immobilization and comfort (i.e., 3% isoflurane in 100% oxygen) which was maintained with 1.5% isoflurane at a constant flow rate of 1 L/min Isoflurane (BioChimico, ItatiaiaRJ, Brazil). Echocardiographic images were acquired when the rat was in the lateral decubitus position.

A MyLabTM30 ultrasound system (Esaote, Genoa, Italy) equipped with a 10 MHz nominal frequency transducer was employed to conduct the assessments. Two-dimensional studies were conducted using a fast-sampling rate of 120 frames per second (fps) in M Mode. Echocardiographic measurements were performed at a sweep speed of 200 mm/s, adjusted in accordance with the heart rate. The evaluation of LV function included ejection fraction (EF) and fractional shortening (FS) (Supplementary Table 1). The flattening of the interventricular septum was assessed (i.e., LV D-shaped).

To characterize PAH, the systolic excursion of the annular plane tricuspid (TAPSE), acceleration time (TA) and ejection time (TE) were used as hemodynamic parameters (Supplementary Table 1). Subsequently, the TA/TE ratio was calculated and used as a pulmonary arterial resistance index.

### Sample collection

The animals in all groups (SC, SH, and EH) were euthanized by decapitation on the day 23 after MCT injection, which represents the median survival time of animals in the SHS group. After euthanasia, the heart and ventricles were dissected, weighed, and processed for analysis as described below.

### Histological analyses and LV morphometry

Histological analyses and LV morphometry were performed as previously described [37]. Briefly, LV sample fragments were collected, fixed in 10% formalin, dehydrated in ethanol, clarified in xylol, and embedded in paraffin. Blocks were cross-sectioned into 5 μm-thick histological sections and subsequently stained with hematoxylin–eosin (H&E) and mounted on histology slides. To avoid repeated analyses of the same histological area, sections were evaluated in a semi-series using one of every 10 sections. Slides were visualized, and images were captured using a light microscope (Olympus BX-50, Tokyo, Japan) connected to a digital camera (Olympus Q Color-3, Tokyo, Japan). Subsequently, analyses of the myocyte cross-sectional area (CSA), total collagen, percentage of cardiomyocytes, and extracellular matrix were performed.

### Antioxidant enzymes and oxidative stress markers

Frozen LV samples of 100 mg were homogenized in 1 mL of phosphate buffer solution at pH 7.4 and then centrifuged at 15,000 × g for 10 min at 4 °C. The resulting supernatant was used to determine the activity of the antioxidant enzymes superoxide dismutase (SOD), catalase (CAT), and glutathione S-transferase (GST), as well as the oxidative metabolite malondialdehyde (MDA) as previously described [38]. Analyses were performed using an ELISA microplate reader (Multiskan GO, Thermo Scientific) or a spectrophotometer (UV-Mini 1240, Shimadzu) as appropriate.

### Isolation of left ventricular myocytes

The heart was connected to a Langendorff retrograde perfusion system, and individual LV myocytes were extracted as previously outlined [34]. In summary, the heart was perfused through the aorta with Tyrode solution composed of the following components: (Sigma-Aldrich, USA) 130 mM NaCl, 1.43 mM MgCl_2_, 5.4 mM KCl, 0.75 mM CaCl2, 5.0 mM HEPES, 10.0 mM glucose, 20.0 mM taurine, and 10.0 mM creatine, at a pH of 7.4, for approximately 5 min. Subsequently, Tyrode’s solution was replaced with a Tyrode’s solution containing EGTA (0.1 mM) for a 6-min period. The heart was then perfused with Tyrode solution enriched with 1 mg/mL collagenase type II (Worthington Biochemical, USA) and 0.1 mg/mL protease (Sigma-Aldrich, USA) for approximately 12 min. Following the digestion process, the LV of the treated heart was excised and cut into small fragments, which were placed in a conical flask containing the enzymatic solution (i.e., collagenase and protease). The cells were mechanically separated by agitating the flasks for 5 min. The dispersed cells were separated from the undispersed tissue by filtration. After centrifugation the isolated cells were immersed in Tyrode solution and stored at 5 °C until needed and were used within 2 to 3 h after isolation. Throughout the isolation procedure, the solutions used were oxygenated with 100% oxygen (White Martins, Brazil) and maintained at a temperature of 37 ºC.

### Measures of myocyte contractility and intracellular calcium transient

The contractile performance of LV myocytes was assessed using an edge detection system (Ionoptix, USA) attached to an inverted microscope (Nikon Eclipse—TS100, Japan) as previously described [33]. Myocytes were positioned in a bath on the stage of the inverted microscope and were continuously exposed to Tyrode’s solution with the following composition (Sigma-Aldrich, USA): 137 mM NaCl, 5.4 mM KCl, 0.33 mM NaH_2_PO_4_, 0.5 mM MgCl_2_, 5 mM HEPES, 5.6 mM glucose, 1.8 mM CaCl_2_, adjusted to pH 7.4 using 5N NaOH, and maintained at a temperature of 37 ˚C. Myocytes were externally stimulated at a frequency of 3 Hz using an electrical stimulator (Myopacer; Field Stimulator, Ionoptix, USA). Only myocytes in good condition with well-defined horizontal borders (i.e., right and left) and sarcomeric striations, relaxed at rest, and without involuntary contractions were used for the experiments.

The intracellular Ca^2+^ transient was assessed according to the established procedures [39]. Briefly, isolated cells were loaded with fura-2 AM (3 mM; Thermo Fisher Scientific, USA). Subsequently, the cells were placed in the experimental chamber and immersed in the same Tyrode’s solution used in the contractility measurement. The cells were then subjected to epi-fluorescence at 37 °C (IonOptix, USA) in a darkened room, while being electrically stimulated (40 V, 5 ms) at a frequency of 3 Hz using a MyoPacer device (IonOptix, USA). The intracellular Ca^2+^ transient was determined by measuring the ratio of the fluorescence emitted at 510 nm in response to excitation at 340 and 380 nm (Fura-2 340:380 nm). The parameters of the intracellular Ca^2+^ transient were determined using the IonWizard 6.3 software (IonOptix, USA).

### Statistical analysis

The data were subjected to the Shapiro–Wilk test to assess their distribution. Parametric data were analyzed using one-way analysis of variance (ANOVA) to evaluate differences between groups, followed by the Tukey’s post-hoc test for multiple comparisons. Non-parametric data were analyzed using the Kruskal–Wallis’ test, followed by the Dunn’s post-hoc test for multiple comparisons, as needed. Quantitative data are presented as mean ± SEM, while qualitative data are presented as percentages. A significance level of up to 5% (alpha error probability) was considered. All data analyses were conducted using the GraphPad Prism Software, version 8.0.2.

## Results

### Animal survival

**Fig. 1:**
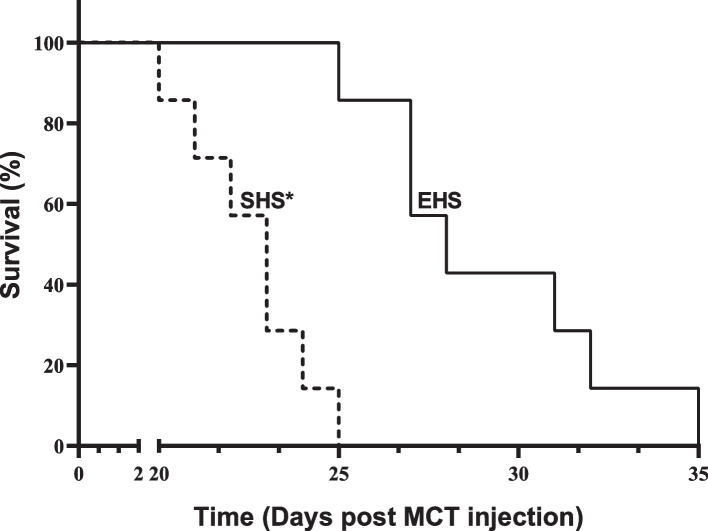
Survival of animals with pulmonary arterial hypertension. SHS, sedentary hypertensive survival. EHS, exercise hypertensive survival. **P < 0.05 vs. EHS. Kaplan–Meier curve, with post-hoc Log-rank test. Data are medians of seven rats in each group

Although all rats in the SHS and EHS experimental groups died, animals in the EHS group had a longer median survival (29 days) than those in the SHS group (23 days) (Fig. 1), which indicates a positive effect of the employed combined physical training.

### Physical effort tolerance

**Fig. 2:**
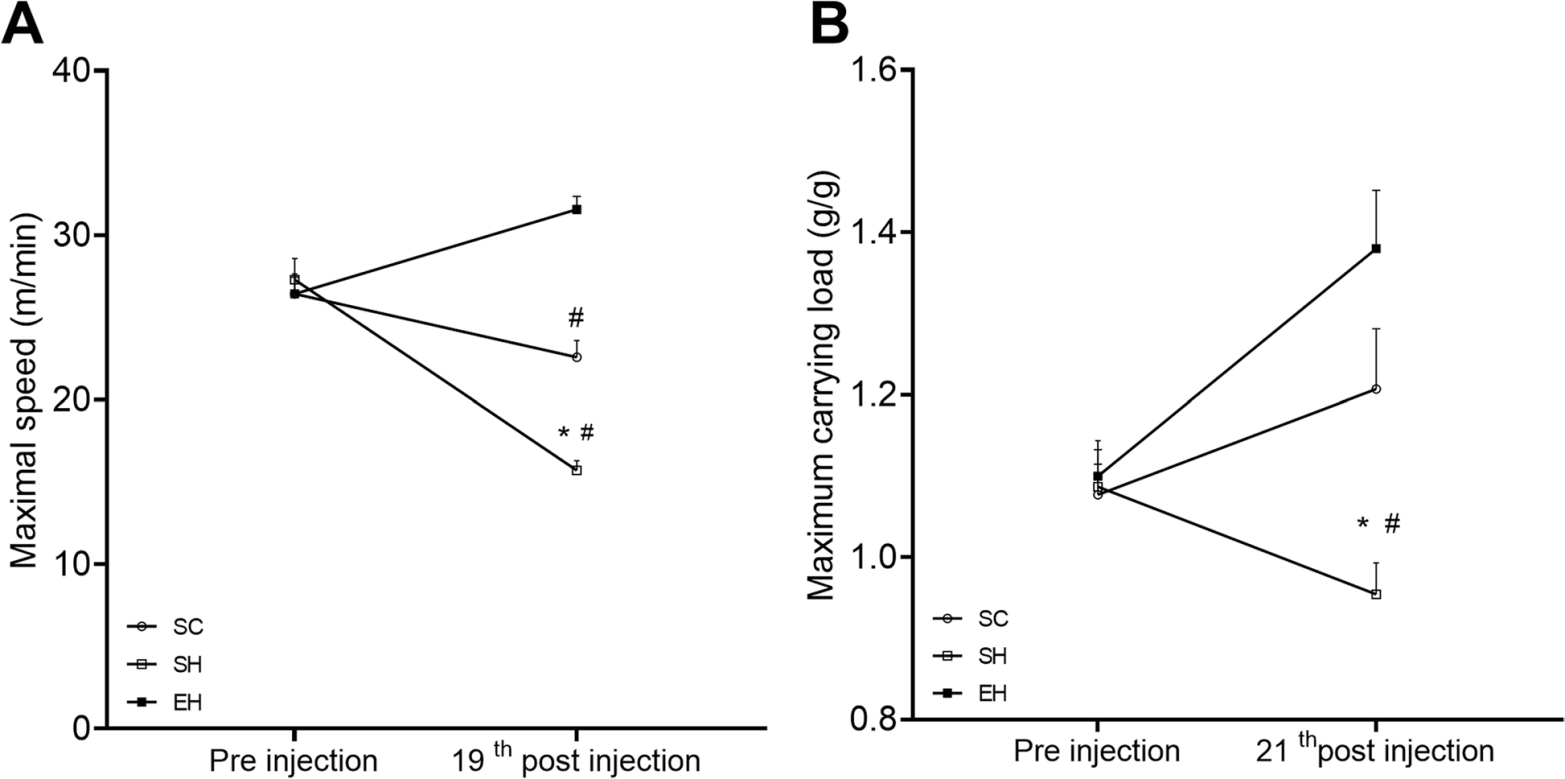
Effects of combined physical training on physical effort tolerance. **A** Maximum speed. **B** Maximum carrying load. Tests performed before (pre-injection), 19 and 21 days after application of monocrotaline. Data are means ± SEM of seven rats in each group. SC sedentary control; SH, sedentary hypertensive; EH, exercise hypertensive. *P < 0.05 vs. SC; # *P* < 0.05 vs. SH. One-way ANOVA followed by Tukey’s post hoc test

There was no difference between the groups before MCT application in terms of MRS (Fig. 2A) or MCL (Fig. 2B). However, 19 and 21 days after injection, the animals in the SH group showed a reduction in physical effort tolerance to both aerobic and resistance exercises compared to those in the SC and EH groups. In addition, animals in the SC group presented a lower MRS than those in the EH group.

### Echocardiographic parameters

**Fig. 3:**
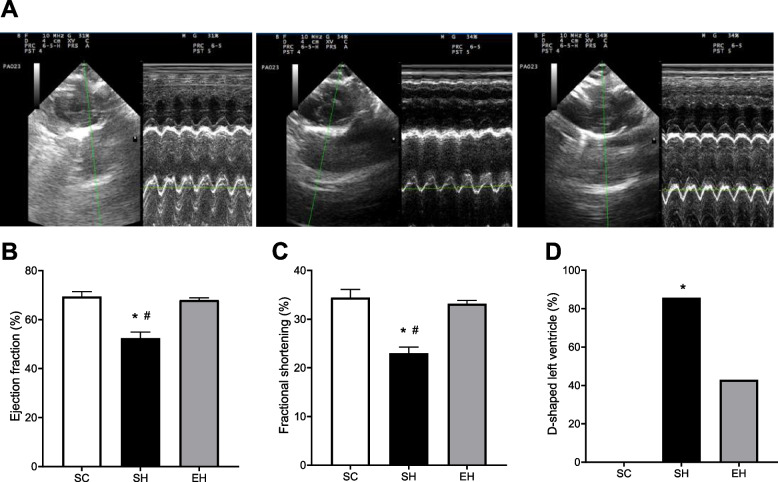
Effects of combined physical training on left ventricular function assessed on day 22 after the first monocrotaline injection. **A** Representative echocardiograph images. **B** Ejection fraction. **C** Fractional shortening. **D** D-shaped left ventricle. Values are means ± SD of seven rats in each group). SC: sedentary control; SH: sedentary hypertensive; EH: exercise hypertensive. Panel **B** and **C** One-way ANOVA followed by the Tukey’s post hoc test. Panel **D** Pearson’ s Chi-squared test. *P < 0.05

On the 22^nd^ day after MCT application, rats in the SH group had lower EF and FS compared to those in the SC and EH groups (Fig. 3B and C). Animals in the SH group also exhibited a greater flattening of the interventricular septum than those in the SC group (Fig. 3D).

The presence of PAH in the SH group was also characterized by the TAPSE values and the TA/TE ratios (Supplementary Table 1). Animals in the SH group presented lower TAPSE values and TA/TE ratios (1.41 ± 0.10 mm and 0.37 ± 0.03, respectively) than those in the SC (2.51 ± 0.33 mm and 0.51 ± 0.02, respectively) and EH (2.22 ± 0.29 mm and 0.51 ± 0.03, respectively) groups.

### Morphometric data

**Fig. 4:**
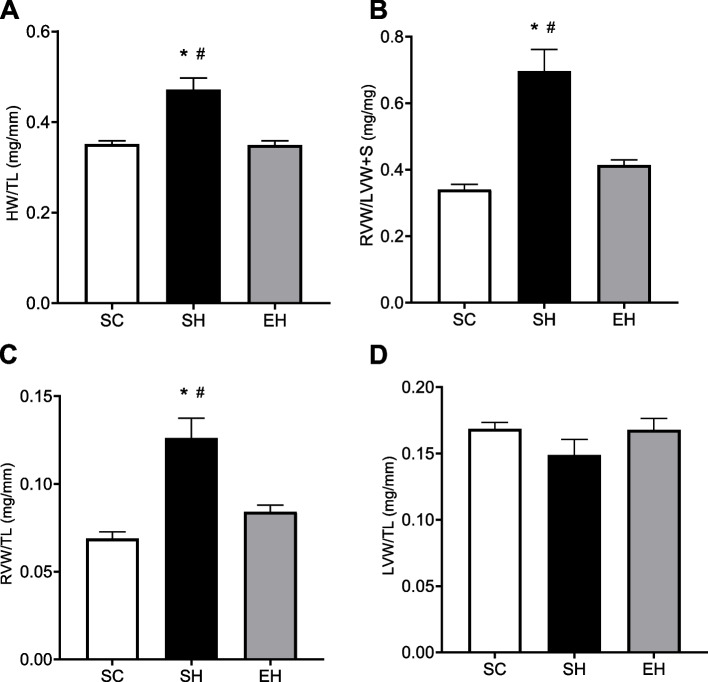
Effects of combined physical training on morphometric parameters. **A** Heart weight/tibial length (**B**) RV/LV + S (**C**) RV weight/tibial length (**D**) LV weight/tibial length. Data are means ± SEM of seven rats in each group. SC, sedentary control; SH, sedentary hypertensive; EH, exercise hypertensive. One-way ANOVA followed by Tukey’s post hoc test. *P < 0.05 vs. SC; # *P* < 0.05 vs. EH

In general, rats in the SH group had a significantly higher right heart hypertrophy index (heart weight/tibial length, RV weight/tibial length, and RV/LV + S) than those in the SC and EH groups (Fig. 4A, B, and C).

### Adverse remodeling

**Fig. 5:**
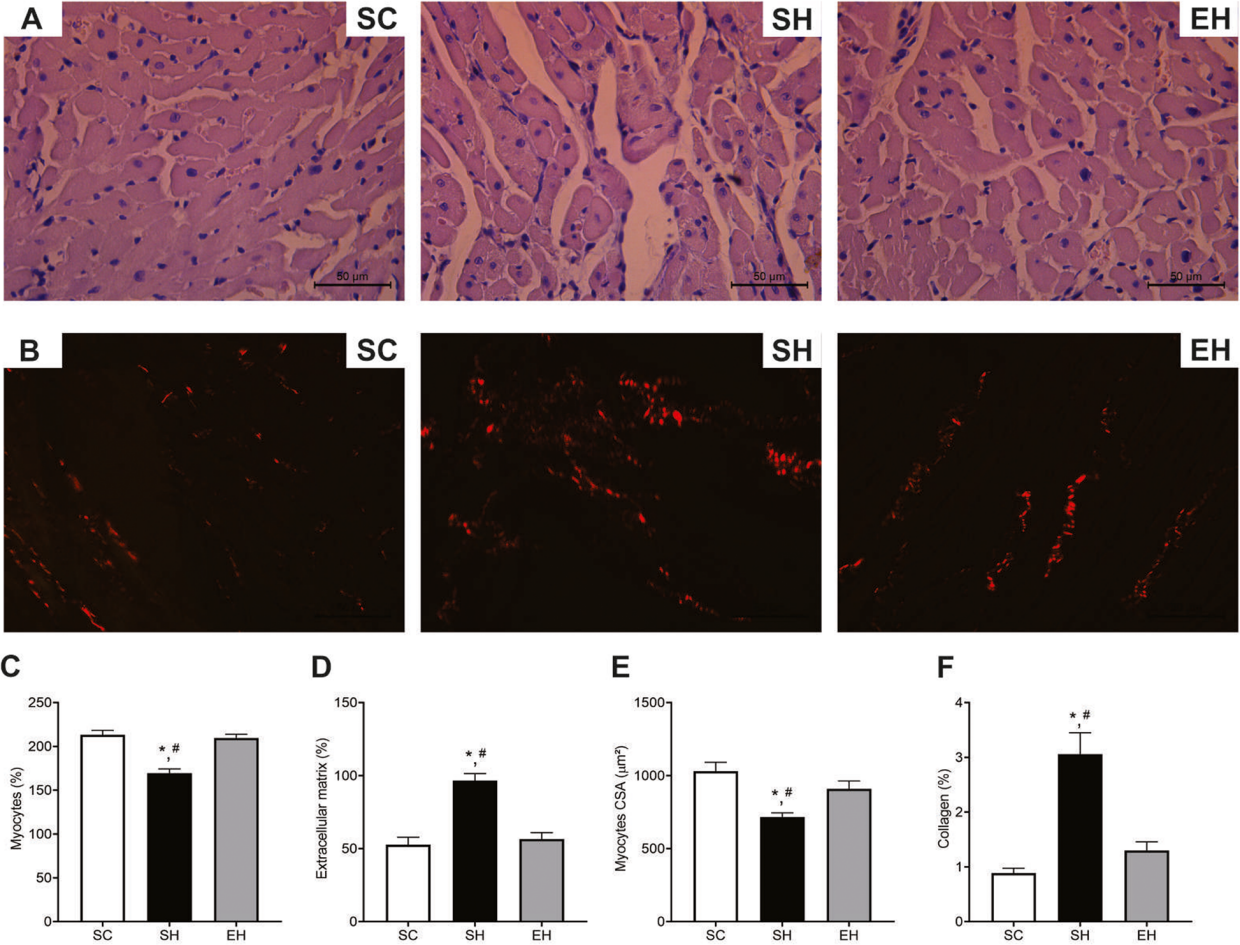
Effects of combined physical training on left ventricle remodeling. **A** Representative photomicrographs of left ventricle tissue (hematoxylin & eosin staining). **B** Representative photomicrographs of collagen in the left ventricle (picrosirius red staining). **C** Percentage of cardiomyocytes. **D** Percentage of extracellular matrix. **E** Cross-sectional area of cardiomyocytes (CSA). **F** Percentage of interstitial collagen. Data are means ± SEM of seven rats in each group. SC, sedentary control; SH, sedentary hypertensive; EH, exercise hypertensive. One-way ANOVA followed by Tukey’s post hoc test. *P < 0.05 vs. SC; # *P* < 0.05 vs. EH

Rats in the SH group presented lower percentage of myocytes (Fig. 5C) and CSA (Fig. 5E) than those in the SC and EH group. However, animals in the SH group exhibited higher percentages of extracellular matrix (Fig. 5D) and total collagen (Fig. 5F) when compared to those in the SC and EH groups.

### Oxidative stress markers

**Fig. 6:**
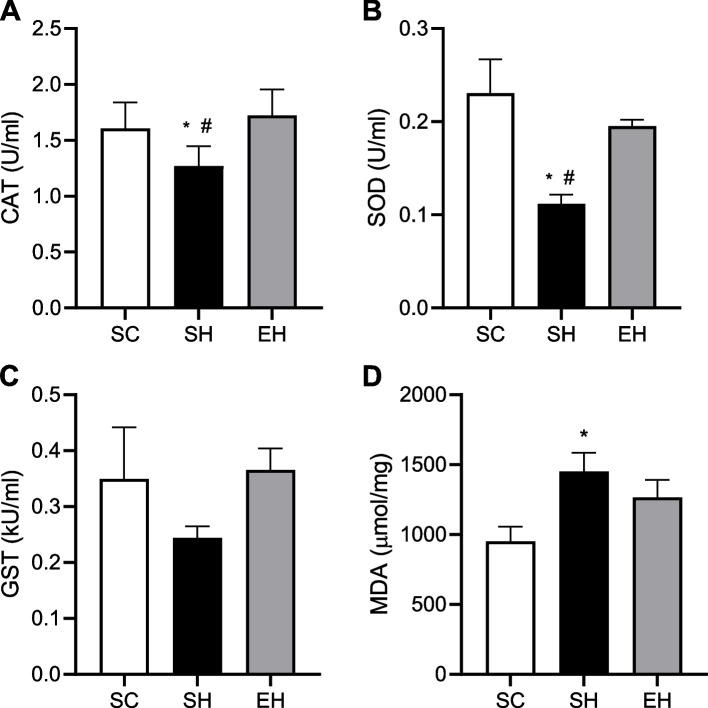
Effects of combined physical training on left ventricular oxidative stress biomarkers. **A** CAT (catalase), **B** SOD (superoxide dismutase), **C** GST (glutathione S-transferase), **D** MDA (malondialdehyde). Data are means ± SEM of six rats in each group. SC, sedentary control; SH, sedentary hypertensive; EH, exercise hypertensive. One-way ANOVA followed by Tukey’s post hoc test. *P < 0.05 vs. SC; # *P* < 0.05 vs. EH, † *P* < 0.05 vs. SH

Rats in the SH group showed lower CAT (Fig. 6A) and SOD (Fig. 6B) activities than those in the SC and EH groups. The activity of GST did not differ between groups (Fig. 6C). Rats in the SH group had higher levels of MDA (Fig. 6D) compared to those in the SC group.

### Myocyte contractile function and intracellular Ca2+ transient

**Fig. 7:**
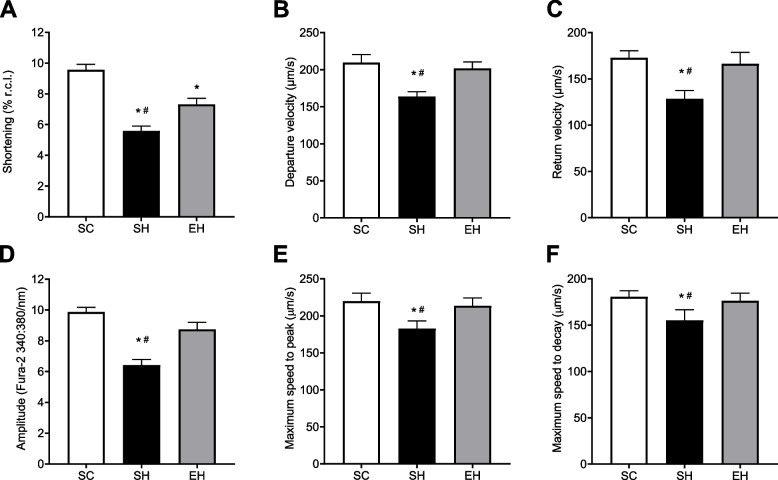
Effects of combined physical training on contractility and intracellular Ca^2+^ transient in single left ventricular myocytes. **A** Amplitude of contraction. **B** Departure velocity of contraction. **C** Return velocity of contraction. **D** Amplitude of the intracellular Ca^2+^ transient. **E** Maximum speed to peak of the intracellular Ca^2+^ transient. **F** Maximum speed to decay of the intracellular Ca^2+^ transient. Data are mean ± SEM of five cells per animal (seven animals per group). Kruskal–Wallis, test followed by Dunn’s test. *P < 0.05 vs. SC; # *P* < 0.05 vs. EH

Myocytes from rats in the SH group presented lower contraction amplitude (Fig. 7A), contraction velocity (Fig. 7B) and relaxation velocity (Fig. 7C), than those from rats in the SC and EH groups. In addition, myocytes from rats in the SH group showed lower amplitude (Fig. 7D), and velocities to peak (Fig. 7E), and to decay (Fig. 7F) of the intracellular Ca^2+^ transient compared to those from rats in the SC and EH groups.

## Discussion

In the present study we examined whether combined physical training of moderate intensity performed during the development of PAH induced by MCT in rats is beneficial to the structure and function of the LV. Our results demonstrate that combined physical training protects the LV from damages induced by MCT to its structure and function.

Initially, the results reveal that the employed exercise regime prolonged the survival of rats injected with MCT. This finding reinforces those reported in this model by our group and elsewhere when rats with MCT-induced PAH were submitted to moderate-intensity aerobic [10, 33, 40] and resistance exercise [12, 20] programs separately starting one day after MCT injection and lasting over the development of the disease. Remarkably, PAH patients submitted to combined exercise interventions exhibit improved survival [29, 30, 41].

Differently from the nonexercised hypertensive rats those submitted to combined exercise training enhanced their tolerance to aerobic and resistance physical effort throughout the experiment. This result strengthens those showed previously in this model when moderate-intensity aerobic [33, 40] and resistance exercise [12, 20] were applied separately. It is conceivable that such exercise benefit is due to prevention of skeletal muscle wasting and dysfunction [12, 19], and to improved cardiac function [12, 20, 33, 40]. Exercise training may also cause positive effects on pulmonary vascular reactivity [42] and pulmonary gas exchange [43].

Our data show that combined exercise promotes benign LV functional and structural adaptations in this experimental model of PAH. For instance, it sustained the cardiac function which was impaired by the MCT injection since it avoided reductions in TAPSE and augments in pulmonary arterial resistance (i.e., TA/TE). Regarding LV, combined exercise impeded reductions in EF and FS, and geometric changes (i.e., D-shaped). At the tissue level, the exercise regime inhibited reductions in the cross-sectional area and percentage of myocytes. Moreover, combined exercise restrained increases in the percentage of extracellular matrix and collagen content, that were enhanced in nonexercised hypertensive rats. Such LV remodeling is in line with the preserved function as it directly influences ventricular filling rate, end-diastolic and systolic volumes, and then stroke volume [17, 18]. Therefore, combined exercise training protected the LV against dysfunction and adverse remodeling which softens the progression of the disease.

Furthermore, we demonstrate here at the cellular level that combined exercise conserved the contractile function of single LV myocytes, which was worsened by MCT. LV myocytes from exercised hypertensive rats presented amplitude and velocities to peak and to decay of the intracellular Ca^2+^ transient as well as the amplitude and velocities to peak and relaxation like those from control rats, whereas these properties were significantly reduced in nonexercised hypertensive rats. MCT-induced PAH is known to reduce myocyte contraction and prolong the velocities of contraction and relaxation in the RV [12, 34, 44] and LV [20]. These contractile dynamics are related mainly to Ca^2+^ cycling and ATPase activity. To illustrate, proteins that regulate Ca^2+^ release (e.g., RyR2) and reuptake (e.g., SERCA2a and p-PLB) from the sarcoplasmic reticulum are reduced in the RV of rats with PAH induced by MCT [12, 40, 45]. Regarding ATPase activity, reduced α/β-MHC ratio was found in the RV of rats with MCT-induced PAH [12]. Thus, it is reasonable that such changes also take place in the LV these rats, though it warrants examination. More important, our combined exercise training shielded LV myocytes from such malignant adaptations during the development of the disease.

We also found in this experimental model that the LV undergoes an increase in the MDA levels while the activities of CAT and SOD are reduced. These results are aligned with previous studies that indicate the association of MCT-induced PAH with augmented cardiopulmonary oxidative stress [8, 9, 11, 46]. More importantly, our combined physical training maintained the activities of CAT and SOD and prevented the increase in MDA levels in the LV of hypertensive rats. These findings indicate that combined exercise saved the LV from oxidative damages caused by MCT. Such benefit helps to explain the positive results achieved on EF, FS, LV adverse remodeling and single myocyte contractile function in exercised hypertensive rats since elevated levels of ROS may induce fibrosis [13], and contractile dysfunction by harming calcium cycling regulatory proteins [14, 15]. Indeed, protective effects of aerobic [10] and resistance exercise [12] training performed separately against oxidative stress have been reported in this experimental model of PAH.

Taken together the LV adaptations presented here show that combined exercise training prevents the damages caused by MCT to the structural and functional properties of the LV. In addition, it induced benefits to the functionality of the right ventricle (i.e., avoidance of TAPSE reduction) and to the pulmonary arterial resistance (i.e., prevention of TA/TE ratio reduction). Such adaptations are indicative of an overall maintenance of the cardiac function to the control levels. Moreover, the employed exercise regime increased the tolerance to physical exertion in animals with PAH. Therefore, such combination of effects may have potentially contributed to softens the progression of the disease and to prolong the animals’ survival.

Finally, this study has limitations. First, here we tested the effects of combined physical training on the LV using only one model of PAH (i.e., MCT-induced PAH). Second, the effects of MCT limit the length of the physical training period (i.e., ~ 3 weeks). Third, in the employed resistance exercise model the speed the rat climbs the ladder cannot be controlled. Forth, the measurement of ejection fraction using M-mode in cases of left ventricular deformation like pulmonary hypertension may not be the most accurate. Despite these limitations, we demonstrated that combined physical training promotes positive effects on the LV structural and functional properties, tolerance to physical effort and survival of rats with MCT-induced PAH.

## Conclusions

We conclude that moderate-intensity combined physical training performed during the development of MCT-induced PAH in rats protects their LV from damages to its structure and function and hence increases their tolerance to physical exertion and prolongs their survival.

### Supplementary Information

**Supplementary Material 1.**

## Data Availability

The datasets used and/or analyzed during the current study are available from the corresponding author on reasonable request.

## Abbreviations

CAT: Catalase
EF: Ejection fraction
EH: Exercise hypertensive
EHS: Hypertensive exercise survival
FS: Fractional shortening
GST: Glutathione S-transferase
LV: Left ventricle
MCL: Maximum carrying load
MCT: Monocrotaline
MDA: Malondialdehyde
MRS: Maximum running speed
PAH: Pulmonary arterial hypertension
PVR: Pulmonary vascular resistance
ROS: Reactive oxygen species
RV: Right ventricle
SC: Sedentary control
SH: Sedentary hypertensive
SHS: Sedentary hypertensive survival
SOD: Superoxide dismutase
TAPSE: Tricuspid annular plane systolic excursion

## References

[1] Humbert M, Guignabert C, Bonnet S, Dorfmüller P, Klinger JR, Nicolls MR (2019). Pathology and pathobiology of pulmonary hypertension: state of the art and research perspectives. Eur Respir J.

[2] Vonk Noordegraaf A, Chin KM, Haddad F, Hassoun PM, Hemnes AR, Hopkins SR (2019). Pathophysiology of the right ventricle and of the pulmonary circulation in pulmonary hypertension: an update. Eur Respir J.

[3] McMurray J, McLay J, Chopra M, Bridges A, Belch JJF (1990). Evidence for enhanced free radical activity in chronic congestive heart failure secondary to coronary artery disease. Am J Cardiol.

[4] Diaz-Velez CR, García-Castiñeiras S, Mendoza-Ramos E, Hernández-López E (1996). Increased malondialdehyde in peripheral blood of patients with congestive heart failure. Am Heart J.

[5] Mathew R, Zeballos GA, Tun H, Gewitz MH (1995). Role of nitric oxide and endothelin-1 in monocrotaline-induced pulmonary hypertension in rats. Cardiovasc Res.

[6] Handoko ML, Schalij I, Kramer K, Sebkhi A, Postmus PE, Van Der Laarse WJ (2008). A refined radio-telemetry technique to monitor right ventricle or pulmonary artery pressures in rats: a useful tool in pulmonary hypertension research. Pflügers Arch.

[7] Hessel MH, Steendijk P, Den Adel B, Schutte CI, Van Der Laarse A (2006). Characterization of right ventricular function after monocrotaline-induced pulmonary hypertension in the intact rat. Am J Physiol Heart Circ Physiol.

[8] Farahmand F, Hill MF, Singal PK. Antioxidant and oxidative stress changes in experimental cor pulmonale. Mol Cell Biochem. 2004;260:21–9. 10.1023/b:mcbi.0000026047.48534.50 15228082

[9] Redout EM, Wagner MJ, Zuidwijk MJ, Boer C, Musters RJ, Van Hardeveld (2007). Right-ventricular failure is associated with increased mitochondrial complex II activity and production of reactive oxygen species. Cardiovasc Res.

[10] Souza-Rabbo MP, Silva LF, Auzani JA, Picoral M, Khaper N, Belló-Klein A (2008). Effects of a chronic exercise training protocol on oxidative stress and right ventricular hypertrophy in monocrotaline-treated rats. Clin Exp Pharmacol Physiol.

[11] Redout EM, Van der Toorn A, Zuidwijk MJ, Van de Kolk CW, Van Echteld CJ, Musters RJ (2010). Antioxidant treatment attenuates pulmonary arterial hypertension-induced heart failure. Am J Physiol Heart Circ Physiol.

[12] Soares LL, Leite LB, Ervilha LOG, Pelozin BRA, Pereira NP, Silva BAF (2023). Resistance exercise training benefits pulmonary, cardiac, and muscular structure and function in rats with stable pulmonary artery hypertension. Life Sci.

[13] Takimoto E, Kass DA (2007). Role of oxidative stress in cardiac hypertrophy and remodeling. Hypertension.

[14] Giordano FJ (2005). Oxygen, oxidative stress, hypoxia, and heart failure. J Clin Investig.

[15] Zima AV, Blatter LA (2006). Redox regulation of cardiac calcium channels and transporters. Cardiovasc Res.

[16] Louie EK, Rich S, Brundage BH. Doppler echocardiography assessment of impaired left ventricular filling in patients with right ventricular pressure overload due to primary pulmonary hypertension. J Am Coll Cardiol. 1986;8:1298–306. 10.1016/s0735-1097(86)80300-x 3782636

[17] Tji-Joong Gan C, Lankhaar JW, Marcus JT, Westerhof N, Marques KM, Bronzwaer JG (2006). Impaired left ventricular filling due to right-to-left ventricular interaction in patients with pulmonary arterial hypertension. Am J Physiol Heart Circ Physiol.

[18] Marcus JT, Noordegraaf AV, Roeleveld RJ, Postmus PE, Heethaar RM, Van Rossum AC (2001). Impaired left ventricular filling due to right ventricular pressure overload in primary pulmonary hypertension: noninvasive monitoring using MRI. Chest.

[19] Correia-Pinto J, Henriques-Coelho T, Roncon-Albuquerque R, Lourenço AP, Melo-Rocha G, Vasques-Nóvoa F (2009). Time course and mechanisms of left ventricular systolic and diastolic dysfunction in monocrotaline-induced pulmonary hypertension. Basic Res Cardiol.

[20] Soares LL, Leite LB, Ervilha LOG, Silva BAFD, Freitas MO, Portes AMO (2022). Resistance exercise training mitigates left ventricular dysfunctions in pulmonary artery hypertension model. Arq Bras Cardiol.

[21] Schmidt C, Bovolini JA, Gonçalves N, Vasques-Nóvoa F, Andrade MDA, Santos M (2020). Exercise preconditioning prevents left ventricular dysfunction and remodeling in monocrotaline-induced pulmonary hypertension. Porto Biomed J.

[22] Lourenço AP, Roncon-Albuquerque R, Brás-Silva C, Faria B, Wieland J, Henriques-Coelho T (2006). Myocardial dysfunction and neurohumoral activation without remodeling in left ventricle of monocrotaline-induced pulmonary hypertensive rats. Am J Physiol Heart Circ Physiol.

[23] Hardziyenka M, Campian ME, Reesink HJ, Surie S, Bouma BJ, Groenink M (2011). Right ventricular failure following chronic pressure overload is associated with reduction in left ventricular mass: evidence for atrophic remodeling. J Am Coll Cardiol.

[24] Lajoie AC, Bonnet S, Provencher S (2017). Combination therapy in pulmonary arterial hypertension: recent accomplishments and future challenges. Pulm Circ.

[25] Zafrir B (2013). Exercise training and rehabilitation in pulmonary arterial hypertension: rationale and current data evaluation. J Cardiopulm Rehabil Prev.

[26] Sahni S, Capozzi B, Iftikhar A, Sgouras V, Ojrzanowski M, Talwar A (2015). Pulmonary rehabilitation and exercise in pulmonary arterial hypertension: an underutilized intervention. J Exerc Rehabil.

[27] Ehlken N, Lichtblau M, Klose H, Weidenhammer J, Fischer C, Nechwatal R (2016). Exercise training improves peak oxygen consumption and haemodynamics in patients with severe pulmonary arterial hypertension and inoperable chronic thrombo-embolic pulmonary hypertension: a prospective, randomized, controlled trial. Eur Heart J.

[28] Pandey A, Garg S, Khunger M, Garg S, Kumbhani DJ, Chin KM (2015). Efficacy and safety of exercise training in chronic pulmonary hypertension: systematic review and meta-analysis. Circulation.

[29] Zhang X, Xu D (2020). Effects of exercise rehabilitation training on patients with pulmonary hypertension. Pulmon Circ.

[30] Yan L, Shi W, Liu Z, Zhao Z, Luo Q, Zhao Q (2021). The benefit of exercise-based rehabilitation programs in patients with pulmonary hypertension: a systematic review and meta-analysis of randomized controlled trials. Pulmon Circ.

[31] Colombo R, Siqueira R, Conzatti A, Lima Seolin BG, Fernandes TRG, Godoy AEG (2016). Exercise training contributes to H2O2/VEGF signaling in the lung of rats with monocrotaline-induced pulmonary hypertension. Vascul Pharmacol.

[32] Nogueira-Ferreira R, Moreira-Gonçalves D, Silva AF, Duarte JA, Leite-Moreira A, Ferreira R (2016). Exercise preconditioning prevents MCT-induced right ventricle remodeling through the regulation of TNF superfamily cytokines. Int J Cardiol.

[33] Silva FJ, Drummond FR, Fidelis MR, Freitas MO, Leal TF, de Rezende LMT (2021). Continuous aerobic exercise prevents detrimental remodeling and right heart myocyte contraction and calcium cycling dysfunction in pulmonary artery hypertension. J Cardiovasc Pharmacol.

[34] Natali AJ, Fowler ED, Calaghan SC, White E (2015). Voluntary exercise delays heart failure onset in rats with pulmonary artery hypertension. Am J Physiol Heart Circ Physiol.

[35] Trueblood NA, Inscore PR, Brenner D, Lugassy D, Apstein CS, Sawyer DB (2005). Biphasic temporal pattern in exercise capacity after myocardial infarction in the rat: relationship to left ventricular remodeling. Am J Physiol Heart Circ Physiol.

[36] Hornberger TA, Farrar RP (2004). Physiological hypertrophy of the FHL muscle following 8 weeks of progressive resistance exercise in the rat. Can J Appl Physiol.

[37] Maarman G, Lecour S, Butrous G, Thienemann F, Sliwa K (2013). A comprehensive review: the evolution of animal models in pulmonary hypertension research; are we there yet?. Pulm Circ.

[38] Souza ACF, Bastos DSS, Sertorio MN, Santos FC, Ervilha LOG, Oliveira LL (2019). Combined effects of arsenic exposure and diabetes on male reproductive functions. Andrology.

[39] Satoh H, Delbridge LM, Blatter LA, Bers DM (1996). Surface:volume relationship in cardiac myocytes studied with confocal microscopy and membrane capacitance measurements: species-dependence and developmental effects. Biophys J.

[40] Moreira-Gonçalves D, Ferreira R, Fonseca H, Padrão AI, Moreno N, Silva AF (2015). Cardioprotective effects of early and late aerobic exercise training in experimental pulmonary arterial hypertension. Basic Res Cardiol.

[41] González-Saiz L, Fiuza-Luces C, Sanchis-Gomar F, Santos-Lozano A, Quezada-Loaiza CA, Flox-Camacho A (2017). Benefits of skeletal-muscle exercise training in pulmonary arterial hypertension: the WHOLEi+ 12 trial. Int J Cardiol.

[42] Kashimura O, Sakai A, Yanagidaira Y (1995). Effects of exercise-training on hypoxia and angiotensin II-induced pulmonary vasoconstrictions. Acta Physiol Scand.

[43] Favret F, Henderson KK, Allen J, Richalet JP, Gonzalez NC (2006). Exercise training improves lung gas exchange and attenuates acute hypoxic pulmonary hypertension but does not prevent pulmonary hypertension of prolonged hypoxia. J Appl Physiol.

[44] Soares LL, Drummond FR, Rezende LMT, Lopes Dantas Costa AJ, Leal TF, Fidelis MR (2019). Voluntary running counteracts right ventricular adverse remodeling and myocyte contraction impairment in pulmonary arterial hypertension model. Life Sci.

[45] Pacagnelli FL, Sabela AKDDA, Mariano TB, Ozaki GAT, Castoldi RC, Carmo EMD, Vanderlei LCM (2016). Fractal dimension in quantifying experimental-pulmonary-hypertension-induced cardiac dysfunction in rats. Arq Bras Cardiol.

[46] De Marco VG, Habibi J, Whaley-Connell AT, Schneider RI, Heller RL, Bosanquet JP (2008). Oxidative stress contributes to pulmonary hypertension in the transgenic (mRen2) 27 rat. Am J Physiol Heart Circ Physiol.

