# Supplementary material for: Combined physical training protects the left ventricle from structural and functional damages in experimental pulmonary arterial hypertension
Source: Clin Hypertens. 2024 May 1;30:12. doi: 10.1186/s40885-024-00270-z (PMC11061945; doi:10.1186/s40885-024-00270-z)
Supplement: Supplementary file 1 — Supplementary Material 1. [file 40885_2024_270_MOESM1_ESM.docx]

**Table 1**- Echocardiographic analysis.

| **GROUP/ANIMAL** | **FE** | **FS** | **TA/TE** | **TAPSE** |
| --- | --- | --- | --- | --- |
| SC - 1 | 73 | 37 | 0,51 | 2,4 |
| SC - 2 | 65 | 31 | 0,56 | 2,6 |
| SC - 3 | 76 | 40 | 0,50 | 2,3 |
| SC - 4 | 67 | 33 | 0,49 | 3,1 |
| SC - 5 | 68 | 33 | 0,53 | 2,6 |
| SC - 6 | 75 | 39 | 0,49 | 2 |
| SC - 7 | 62 | 28 | 0,50 | 2,6 |
| **Mean ± SEM** | 69,43 ± 2,01 | 34,43 ± 1,66 | 0,51 ± 0,01 | 2,51 ± 0,13 |
| SH - 1 | 44 | 18 | 0,37 | 1,5 |
| SH - 2 | 47 | 20 | 0,37 | 1,4 |
| SH - 3 | 55 | 25 | 0,43 | 1,3 |
| SH - 4 | 55 | 25 | 0,39 | 1,4 |
| SH - 5 | 48 | 21 | 0,35 | 1,4 |
| SH - 6 | 55 | 25 | 0,38 | 1,6 |
| SH - 7 | 63 | 27 | 0,30 | 1,3 |
| **Mean ± SEM** | 52,43 ± 2,45 | 23,00 ± 1,25 | 0,37 ± 0,01 | 1,41 ± 0,04 |
| EH - 1 | 64 | 30 | 0,50 | 2 |
| EH - 2 | 69 | 34 | 0,46 | 2,2 |
| EH - 3 | 72 | 36 | 0,52 | 2,2 |
| EH - 4 | 68 | 33 | 0,52 | 2,2 |
| EH - 5 | 68 | 33 | 0,55 | 2,6 |
| EH - 6 | 68 | 34 | 0,57 | 2,6 |
| EH - 7 | 67 | 32 | 0,49 | 1,8 |
| **Mean ± SEM** | 68,00 ± 0,90 | 33,14 ± 0,70 | 0,52 ± 0,01 | 2,23 ± 0,11 |

Individual data from each animal in their respective groups and the means ± SEM of 7 rats in each group. SC, sedentary control; SH, hypertensive sedentary; EH, hypertensive exercise. FE, Ejection fraction; FS, Fractional shortening; TA/TE, Acceleration time (TA)/ejection time (TE) ratio; TAPSE, Tricuspid annular plane systolic excursion.
